# Potentially inappropriate medications in older patients based on Beers criteria: a cross-sectional study of a family medicine practice in Saudi Arabia

**DOI:** 10.3399/bjgpopen20X101009

**Published:** 2020-02-05

**Authors:** Atheer Alturki, Tareef Alaama, Yousef Alomran, Ahmed Al-Jedai, Hajer Almudaiheem, Ghassan Watfa

**Affiliations:** 1 Family Medicine Specialist, King Saud Medical City, Central Health Cluster One, Riyadh, Kingdom of Saudi Arabia; 2 Deputy Minister for Therapeutic Affairs, Ministry of Health, Deputyship of Therapeutic Affairs, Riyadh, Kingdom of Saudi Arabia; 3 Assistant Professor and Consultant of Internal Medicine & Geriatric Medicine, King Abdulaziz University, Jeddah, Kingdom of Saudi Arabia; 4 Consultant of Family Medicine and Associate Executive Director for Primary Care and Community Health, King Saud Medical City, Central Health Cluster One, Riyadh, Kingdom of Saudi Arabia; 5 Assistant Deputy Minister for Medical Support Services, Ministry of Health, Deputyship of Therapeutic Affairs, Riyadh, Kingdom of Saudi Arabia; 6 Professor and Consultant Clinical Pharmacist, Solid Organ Transplant, College of Medicine and Pharmacy, Alfaisal University, Riyadh, Kingdom of Saudi Arabia; 7 Drug Policy Department Manager, Ministry of Health, Deputyship of Therapeutic Affairs, Riyadh, Kingdom of Saudi Arabia; 8 Consultant of Geriatric Medicine and Director of Home Health Care Administration, King Saud Medical City, Central Health Cluster One, Riyadh, Kingdom of Saudi Arabia

**Keywords:** Saudi Arabia, family medicine, potentially inappropriate medications, Beers criteria, geriatrics, aged

## Abstract

**Background:**

The use of potentially inappropriate medications (PIMs) is an important issue in older patients who are at risk of adverse drug events.

**Aim:**

To determine the prevalence of PIM use, according to Beers criteria, among an older population (aged ≥65 years) in a large family medicine setting, and to identify the associated risks.

**Design & setting:**

A prospective cross-sectional study of patients aged ≥65 years was conducted from June 2017 to June 2018 at the Family and Community Medicine (FCM) clinics of King Saud Medical City (KSMC) in Riyadh, Saudi Arabia.

**Method:**

This cross-sectional study included patients aged ≥65 years who were seen at new appointments or followed-up at the FCM clinics of KSMC in Riyadh, Saudi Arabia. Data were collected by extensive face-to-face interviews and from the patients’ medical records.

**Results:**

A total of 270 older patients aged 72.41 ±6.23 years (mean ±standard deviation [SD]) were included in the present study. The prevalence of PIMs was 60.7% (*n* = 164). Multivariate analyses identified three independent variables associated with PIMs: incremental age per 5 years (odds ratio [OR] 1.47, 95% confidence intervals [CI] = 1.15 to 1.88; *P* = 0.002), female sex (OR 1.95, 95% CI = 1.10 to 3.42; *P* = 0.021), and polypharmacy (OR 8.21, 95% CI = 4.58 to 14.7; *P*<0.001). The most common PIMs used were 39.4% related to proton pump inhibitors (PPI), 25.2% to diuretics (other than spironolactone), 10.6% to non-steroidal anti-inflammatory drugs (NSAIDs), and 8.7% to aspirin use.

**Conclusion:**

This study showed high prevalence of PIMs. Increasing age, female sex, and polypharmacy were found to be significant risk factors for PIM use. The frequency of morbidities was not significantly different among patients with PIMs compared to those without PIMs, except for hypertension and osteoarthritis, which were more common in the PIMs group. The present study reinforces the importance of comprehensive medication management and reviews.

## How this fits in

PIM usage is a problem among older patients who are at risk of adverse drug events. This study, conducted in Saudi general practice, showed a highly prevalent use of PIMs (60.7%, *n* = 164), which is higher than the rates previously reported in western countries, and even higher than the previous studies from Saudi Arabia. The present study reinforces the importance of conducting comprehensive medication management for older patients in general practice to avoid any potential harm related to PIMs.

## Introduction

Inappropriate medication usage is an issue among older patients (aged ≥65 years), particularly those who have concomitant morbidities. They are more likely to experience drug-related adverse events than younger populations.^1,2^ Drugs are usually given to older patients based on the efficacy and safety studies conducted among younger patients who do not have similar morbidities.^3,4^ Causation of drug-related adverse events in older patients is multifactorial, including complex medication regimens, ageing, and changes in medicine pharmacokinetics and pharmacodynamics.^5^

It has been reported that the adverse drug events related to PIMs are associated with the rate of hospitalisation.^6^ The use of inappropriate medications in this population has been shown to cause approximately 5% of hospital admissions.^7^ Moreover, a further study has shown interacting medications leading to adverse drug reactions in up to 35% of older outpatients, and 40% of inpatients with inappropriate medication use.^8^ The Beers criteria were developed by Mark H Beers in 1991 at the University of California, Los Angeles, with subsequent updates. It was adopted later by the American Geriatrics Society, which recently updated the criteria in 2015. It is used to assess medication safety among older patients.^9^ It highlights the medications to be avoided in older patients, as well as the medications that should be avoided in certain clinical conditions or concomitantly with other interacting medications and those to be used with caution in this sensitive age group.^10^ The older patient population is vulnerable to physical impairments and health problems. They are more likely to endure chronic health conditions, deficiencies, digestive problems, impaired metabolism and excretion, and susceptibility to adverse drug effects.

The demographic of the Saudi population is changing. It is a growing population with an increasing life expectancy, and a high prevalence of chronic diseases. In 2015, 3.1% of the Saudi Arabian population was aged ≥65 years. Projections show that the proportion of the older population (aged ≥65 years) will continue to increase and will eventually reach 6.6% of the total Saudi population in 2030 and 16.7% by 2050.^11^

In Canada, a higher proportion of females (42.0%) than males (31.0%) were shown to have filled PIM prescriptions.^12^ Family doctors were found to prescribe sedatives and/or hypnotics more frequently in older patients for many reasons: limited knowledge regarding PIMs; limited applicability of PIMs lists in daily practice; lack of time; and lack of therapeutic alternatives. Other patient-related factors that also affected family doctors’ prescribing practice include: bad experiences regarding changes of medication; refusal to follow prescriptions of sedative and/or hypnotics with doses that are not considered PIMs; and refusal to stop the prescribed sedatives and/or hypnotics if necessary. This resulted in some form of resignation on the physician’s side.^13^ An Italian study showed a lower percentage of PIM use (28.0%), and that females were more likely to be exposed to PIMs than males.^14^ In Taiwan, the prevalence of having at least one PIM was reported to be high at 82.67%, and these were mostly prescribed by internist or family physicians and neurologists or psychiatrists.^15^

The present study was conducted to determine the prevalence of PIM use among older patients (aged ≥65 years) in a family medicine setting in Saudi Arabia based on the Beers 2015 criteria, and to identify the factors associated with PIMs.

## Method

This cross-sectional study was conducted from 1 June 2017–31 May 2018 at the FCM clinics of KSMC, a 1351-bed tertiary care hospital in Riyadh, Saudi Arabia. The FCM clinics are a set of clinics serving a catchment area of multiple primary health care centers (PHCs). The FCM clinics are based in a tertiary care institute and receive patients that are referred from PHCs. Hence, patients seen at the clinics had medical needs that could not be solved at the level of the PHC and needed more resources or capabilities. Family physicians see patients referred from PHCs on an appointment basis as new referrals, and further follow-up appointments are scheduled if needed. The present study included all patients aged ≥65 years who were seen by the family physicians at KSMC FCM clinics with new appointments or as follow-up. Patients who had undergone organ transplantation were excluded from the study due to their inevitably high polypharmacy. Only patients with medical records at KSMC were invited for study participation, as FCM clinics also receive patients outside of the catchment area for whom medications are not well documented.

Non-probability convenience sampling was used to recruit participants. Older patients were invited and informed about the purpose of the study. Written informed consent was collected from all consenting patients and from Healthcare Power of Attorney for patients with severe dementia, or patients who were not capable of making decisions on their own.

Data were collected using a case report form, which included demographic profiles of the patients (age and sex). Clinical data were additionally collected by extensive face-to-face interviews and from the patients’ medical records.

During face-to-face interviews, all patients underwent clinical evaluations, providing a verbal medical history to the interviewer, which was subsequently verified against the patients’ medical records. The presence of any morbidity was classified into two categories ’Yes‘ and ’No’. The results of biological analyses were captured from the patients’ medical records.

Information on the nature of any currently prescribed drugs was collected from the patients’ medical records and classified into two categories, ’Yes‘ and ’No‘ (medications were identified by their pharmacological class). The number of drugs taken on a daily basis and long-term basis were also recorded. Polypharmacy was evaluated with a binary parameter as the consumption of ≥4 different drugs chronically on a daily basis.^16^ The American Geriatrics Society 2015 Updated Beers Criteria^9^ were used to determine PIMs use. All collected data were entered on the study case report form.

### Statistical analysis

Sample size was calculated using the Metcalfe formula.^17^ Assuming 52.5% prevalence of PIMs in older patients,^7^ and with 6% margin of error, 80% power, and 95% CI, the calculated sample size was 270 patients.

All analyses were carried out using the Statistical Package for Social Sciences (version 23). Univariate analyses were performed after individuals were stratified into two groups according to PIMs use; χ² tests were performed when comparing categorical variables between groups; and *t*-tests were used for continuous variables, as the sample distribution is normal. The significance of the results is represented as a *P* value. A *P* value of <0.05 was considered statistically significant. Results are presented as mean ±SD for quantitative variables and as a percentage for categorical variables.

Logistic regression model analysis was performed with an interactive backward selection method in order to develop a model including parameters associated with PIM use. Regression coefficients, ORs, and 95% CIs were calculated. Validity of the model assumption was verified using analysis of model residuals and testing for heteroscedasticity.

## Results

Among the 282 patients invited to take part in the study, 12 patients did not agree to participate (95.7% acceptance rate). A total of 270 older patients were included in the study (143 female, 53.0%; mean age 72.41 ±6.23 years). There were 77 patients (28.5%) with one morbidity, 109 (40.4%) with two morbidities, and 81 (30.0%) with >2 morbidities. Three patients had no comorbidities. There were 164 patients (60.7%) who reported having used PIMs: three patients had four PIMs (1.8%); 17 patients had three PIMs (10.4%); 34 patients had two PIMs (20.7%); and the remaining 110 patients had only one PIM (67.1%).

Table 1 shows the demographic variables, and the common chronic diseases of the population study, as well as a comparison between patients with and without PIMs.

**Table 1.** Demographics and frequency of morbidities

Parameter	All patients	PIM^a^	*P* value^b^
Yes	No
Total	270	164	106	
Mean age, years ±SD	72.41 ±6.23	73.09 ±6.68	71.35 ±5.33	0.024
Female sex, *n* (%)	143 (52.9)	95 (57.9)	48 (45.3)	0.042
Mean number of medications ±SD	4.09 ±2.19	4.84 ±2.09	2.88 ±1.76	<0.001
Polypharmacy, *n* (%)	149 (55.2)	119 (72.6)	30 (28.3)	<0.001
Mean number of morbidities ±SD	2.11 ±1.14	2.16 ±0.95	2.04 ±1.39	0.397
Hypertension, *n* (%)	196 (72.6)	130 (79.3)	66 (62.3)	0.002
Diabetes mellitus, *n* (%)	146 (54.1)	91 (55.5)	55 (51.9)	0.562
Dyslipidaemia, *n* (%)	59 (21.9)	38 (23.2)	21 (19.8)	0.540
Low back pain, *n* (%)	15 (5.6)	9 (5.5)	6 (5.7)	0.952
Heart failure, *n* (%)	11 (4.1)	9 (5.5)	2 (1.9)	0.144
Stroke, *n* (%)	9 (3.3)	6 (3.7)	3 (2.8)	0.711
Chronic kidney disease, *n* (%)	4 (1.5)	2 (1.2)	2 (1.9)	0.658
Diabetic nephropathy, *n* (%)	2 (0.7)	1 (0.6)	1 (0.9)	0.755
Osteoporosis, *n* (%)	20 (7.4)	13 (7.9)	7 (6.6)	0.675
Osteoarthritis, *n* (%)	15 (5.6)	13 (7.9)	2 (1.9)	0.034
Bronchial asthma, *n* (%)	9 (3.3)	5 (3.0)	4 (3.8)	0.735
Hyperuricaemia, *n* (%)	2 (0.7)	1 (0.6)	1 (0.9)	0.755
Hypothyroidism, *n* (%)	21 (7.8)	11 (6.7)	10 (9.4)	0.414
Anaemia, *n* (%)	4 (1.5)	2 (1.2)	2 (1.9)	0.658
BPH, *n* (%)	22 (8.1)	12 (7.3)	10 (9.4)	0.535

BPH = benign prostatic hypertrophy. PIM = potentially inappropriate medication.

^a^Patients taking ≥1 PIMs. ^b^Probability of the *t*-test (continuous variables) or χ² tests (categorical variables).

The average number of morbidities in the cohort was 2.11 ±1.14 morbidities. The prevalence of hypertension and diabetes was high among participants (72.6% and 54.1%, respectively). Polypharmacy was recorded at 25.2% and was significantly more common among patients with PIMs than those without PIMs. Moreover, patients with PIMs had higher hypertension prevalence than patients without PIMs (*P* = 0.002), as shown in Table 1. Patients with PIMs were also more frequently treated with beta-blockers, calcium channel blockers (CCBs), and angiotensin-converting enzyme (ACE) inhibitors (Table 2).

**Table 2.** Frequency of medications used

Medication	All patients, *n* (%) (*n* = 270)	PIMs, *n* (%)^a^	*P* value^b^
Yes, *n* (%) (*n* = 164)	No, *n* (%) (*n* = 106)
Metformin	116 (43.0)	74 (45.1)	42 (39.6)	0.373
Sulfonylureas	74 (27.4)	44 (26.8)	30 (28.3)	0.791
DPP4 Inhibitors	28 (10.4)	18 (11.0)	10 (9.4)	0.685
TZDs	2 (0.7)	1 (0.6)	1 (0.9)	0.755
Beta-blockers	30 (11.1)	24 (14.6)	6 (5.7)	0.022
CCBs	92 (34.1)	64 (39.0)	28 (26.4)	0.033
ACE inhibitors	67 (24.8)	51 (31.1)	16 (15.1)	0.006
ARBs	50 (18.5)	33 (20.1)	17 (16.0)	0.399
Clopidogrel	17 (6.3)	13 (7.9)	4 (3.8)	0.170
PPIs	94 (34.8)	91 (55.5)	3 (2.8)	<0.001
NSAIDs	55 (20.4)	35 (21.3)	20 (18.9)	0.622
Aspirin	112 (41.5)	82 (50.0)	30 (28.3)	<0.001
Thyroxine	22 (8.1)	11 (6.7)	11 (10.4)	0.282
Statins	141 (52.2)	97 (59.1)	44 (41.5)	0.005
Antibiotics	5 (1.9)	2 (1.2)	3 (2.8)	0.338
Antihistamines	23 (8.5)	20 (12.2)	3 (2.8)	0.007
Bisphosphonates	12 (4.4)	8 (4.9)	4 (3.8)	0.667
Teriparatide	3 (1.1)	1 (0.6)	2 (1.9)	0.328
Tolterodine	1 (0.4)	0 (0.0)	1 (0.9)	0.213
Tamsulosin	20 (7.4)	10 (6.1)	10 (9.4)	0.307
Finastride	11 (4.1)	5 (3.0)	6 (5.7)	0.289

^a^Patients taking ≥1 PIMs. ^b^Probability of χ² tests (categorical variables).

ACE angiotensin-converting enzyme ARBs angiotensin receptor blockers CCBs calcium channel blockers DPP4 dipeptidyl peptidase-4 NSAIDs non-steroidal anti-inflammatory drugs PIMs potentially inappropriate medications PPIs proton pump inhibitors TZDs thiazolidinediones

Compared to patients without PIMs, patients with PIMs were significantly older (73.09 ±6.68 years versus 71.35 ±5.33 years; *P* = 0.024) with a higher proportion of females (*n* = 95/164, 57.9% versus *n* = 69/164, 42.1%; *P* = 0.042). The frequency of morbidities was not significantly different among patients with PIMs compared to those without PIMs; however, significant use of PIMs was found in patients with hypertension and osteoarthritis (*P* = 0.002 and *P* = 0.034, respectively) (Table 2).

There were no documented cases of chronic obstructive pulmonary disease, gastroesophageal reflux disease, vitamin D deficiency, or prostate cancer among patients with PIMs, and only one case of each disease in the patient group without PIMs (data not shown).

The number of medications was significantly higher among patients with PIMs than those without PIMs (4.84 ±2.09 versus 2.88 ±1.76; *P*<0.001), with higher polypharmacy prevalence in the PIMs subgroup compared to those without PIMs (72.6% and 28.3%, respectively; *P*<0.001) (Table 1).

The two subgroups did not differ significantly in terms of overall biological parameters, except for the potassium level, which was lower in the PIMs subgroup (4.42 ±0.55 versus 4.59 ±0.54 mmol/L; *P* = 0.021; data not shown).

Table 2 shows the medications used by all patients and a comparison between patients with and without PIMs. Patients in the PIMs subgroup were more frequently treated with CCBs (*P* = 0.033), ACE inhibitors (*P* = 0.006), PPIs (*P*<0.001), aspirin (*P*<0.001), statins (*P*<0.001), and antihistamines (*P* = 0.007).

Among patients with PIMs, 58 patients received diuretics; one methyldopa; one alpha-blockers; one corticosteroids; two hydroxychloroquine; two sulfasalazine; one betahistine; two methotrexate; seven mebeverine; two antiemetics; two tricyclic antidepressants; seven anticonvulsants; four vasodilators; two serotonin–norepinephrine reuptake inhibitors; one memantine; two levodopa; one muscle relaxant; one rivaroxaban; three isosorbide dinitrate; and seven allopurinol among patients without PIM use. There was no documented use of these medications among patients without PIMs. By contrast, one patient used tolterodine in the PIMs subgroup versus no patients in the subgroup without PIMs (data not shown).

Concerning the frequencies of the most common PIMs in the population study as per the Beers criteria, 39.4% were related to PPIs, 25.2% to diuretics (other than spironolactone), 10.6% to NSAIDs, and 8.7% to aspirin.

**Figure 1.**
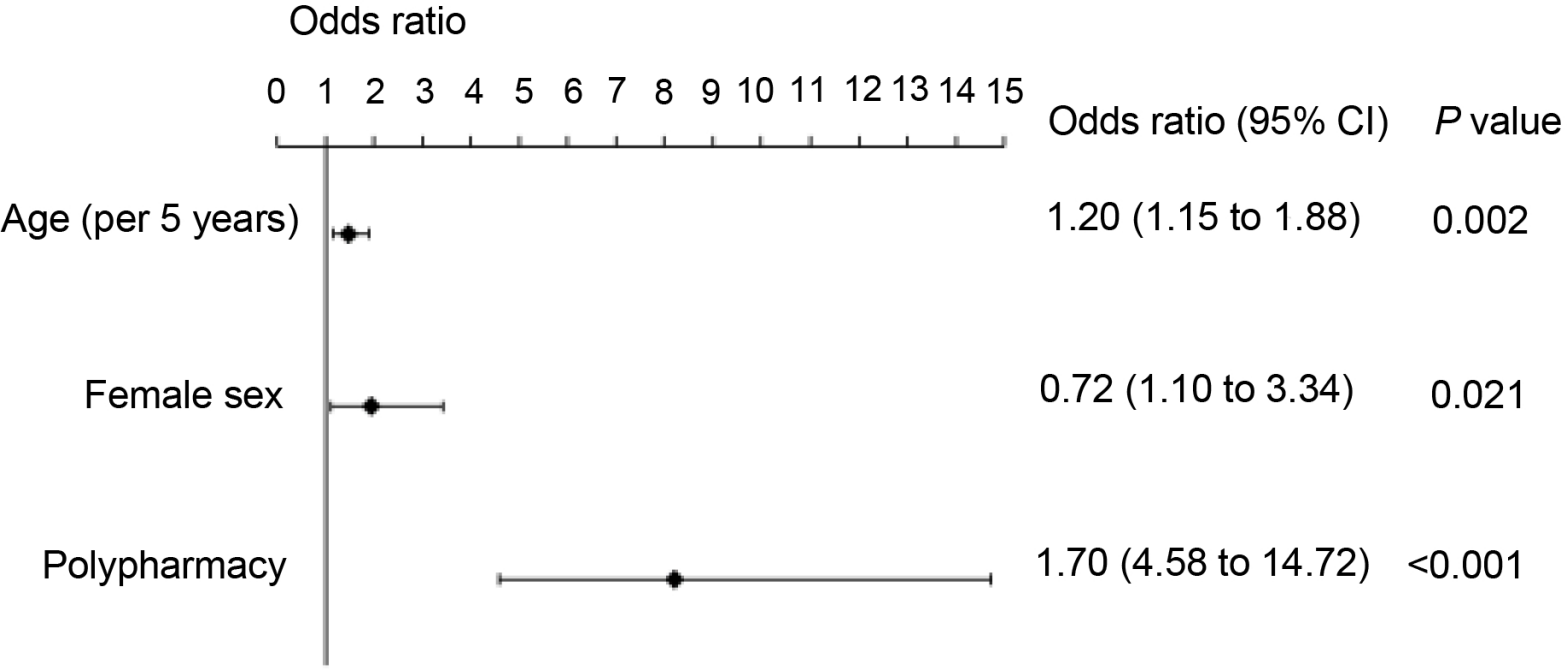
Analysis of factors associated with potentially inappropriate medication using a multiple logistic regression model

In order to identify independent factors for PIMs, analyses were conducted using multiple logistic regression (Figure 1), including and controlling for all variables, revealing differences under univariate analysis. The multivariate analysis identified three independent parameters associated with PIMs: incremental age per 5 years (*r* = 0.39 ±0.12; *P* = 0.002), female sex (*r* = 0.66 ±0.29; *P* = 0.021), and polypharmacy (2.11 ±0.29; *P*<0.001). The model accounted for 46% of the total variance in PIMs.

## Discussion

### Summary

This study showed a highly prevalent use of PIMs (60.7%, *n* = 164). Incremental age per 5 years, female sex, and polypharmacy were identified as independent parameters associated with PIMs. Oral antidiabetic agents (long-acting sulfonylureas), PPIs, diuretics, NSAIDs, and aspirin were the most commonly used PIMs.

### Strengths and limitations

Although the Saudi population is a growing one, with a projected increased life expectancy^11^ and high prevalence of chronic diseases, there are few studies concerning the use of PIMs in this population. The strength of the present study, compared to the currently available data from retrospective studies, is that it was conducted prospectively in a large general practice setting.

One limitation of the study was that the authors were not able to verify adherence to medications. Without this limitation, the authors are confident that the present study would have provided a better insight into the use of PIMs in general practice in Saudi Arabia.

Excluding hypertension and osteoarthritis, the frequency of morbidities was not significantly different among patients with PIMs compared to those without PIMs; any interpretation in this regard should be made with caution given the small subgroup sizes, which may have led to an underpowered statistical comparison. The present study had no missing data, except for some blood analysis.

### Comparison with existing literature

The rate of PIMs (60.7%, *n* = 164) is higher than the rates previously reported in the US, Canada, and Italy, and even higher than previous studies in Saudi Arabia that reported high rates of PIM use.^7,12,14,18^ Previous studies conducted in Saudi Arabia showed PIMs rates of 52.5%^7^ and 2.1%–2.5%.^19^ Differences in the tested populations of these studies may have contributed to the discrepancy between the reported results from previous studies and those obtained herein, since the present study was conducted in a family medicine setting; in Al Odhayani *et al*,^7^ 798 older patients were arbitrarily selected from Prince Sultan Medical Military City through the patient register from the family medicine chronic disease clinics in the Home Health Care programme; in Al-Omar *et al*,^19^ the authors conducted a retrospective cross‐sectional study of outpatient pharmacy prescription records of older patients at Riyadh Military Hospital.

In 2017, the general authority for statistics in Saudi Arabia conducted the *Elderly Survey*,^20^ showing that 4.19% of the total Saudi population are aged ≥65 years, with the female population representing 51.1% of the older population. This older population survey showed high prevalence of hypertension (28.5%) and diabetes mellitus (28.7%) among the older Saudi population. A retrospective cross-sectional study^21^ targeting 3009 older Saudi patient profiles revealed that 55% were suffering from polypharmacy, with an average of 6.4 medications prescribed for patients aged 65–70 years, and an average of 4.2 medications prescribed for those aged ≥71 years.

The present study identified incremental age per 5 years, female sex, and polypharmacy as independent parameters associated with PIMs (Figure 1). These findings are similar to what has been reported in previous studies. In a large study conducted in an outpatient setting, female sex and polypharmacy were significantly associated with PIM prescribing.^22^ A further study showed that PIM use increased considerably with older age.^23^

Previous studies have reported that polypharmacy was associated with poor health outcomes, particularly when used by the older patient population.^4,10^ Findings in the present study are consistent with previous literature that identified polypharmacy as a risk factor for PIM use.^4,24–26^ This is particularly true among the older population when these patients were identified to have received PIMs in the emergency room, where patients come with multiple morbidities.^24–26^ Because of this, the treating physician, together with the pharmacist, is in the perfect position to identify patients who are potential users of PIMs and provide evidence-based guidelines and recommendations to circumvent overuse of medications.

The present study highlights that female sex, independent of polypharmacy, was a significant risk factor for PIMs. These results are supported by previous studies that showed polypharmacy and female sex to be important determinants for an increased likelihood of receiving a PIM prescription.^27–29^

Findings in the present study confirm the high prevalence of both hypertension and diabetes among the older Saudi population (72.6% and 54.1%, respectively). Such a high prevalence of hypertension and diabetes was anticipated because the enrolment was not from the community (that is, healthy population). The FCM clinics are based in a medical city (a tertiary care institute) and receive patients referred from PHCs. Supriya *et al* reported that older hypertensive patients encountered more PIMs;^30^ however, neither hypertension nor anti-hypertensive drugs were identified as independent factors associated with PIMs in the multivariate logistic regression model (Figure 1).

The present study showed that oral antidiabetic agents (long-acting sulfonylureas), PPIs, diuretics, NSAIDs, and aspirin are the most commonly used PIMs. The other recorded PIMs, with lower use frequencies, were hydralazine, ranitidine, chlorpheniramine, amitriptyline, nifidipine, methyldopa, metoclopramide, pergabaline, and prazosin. The strength of recommendation was strong for all of the above PIMs excluding hydralazine, which was weak following Beers criteria.^9^

The Beers criteria recommend avoiding the use of oral long-acting sulfonylureas (glyburide) since its inappropriate use in older adults was found to be significantly associated with a high risk of severe prolonged hypoglycaemia.^9,31^

As mentioned in the results section, all 58 patients who used diuretics were included in the PIMs group. Beers criteria recommend caution with their use, and stress the need to monitor sodium level.^9^ Diuretics are usually the recommended first-line antihypertensive drug for older patients; however, the risk of developing hyponatraemia in older patients is high, particularly among females (OR 3.10), compared to younger patients with hypertension.^32^ Dehydration and electrolyte disturbances also occur as a result of overuse or inappropriate use of diuretics.^33^

In the present study, 86 patients from the 94 treated by PPIs were recorded as using PIMs since Beers criteria recommended avoiding PPI use for >8 weeks unless a clear indication exists.^9^ Indeed, long-term or inappropriate use of PPIs may lead to severe hypochlorhydria, which leads to bacterial colonisation and increased susceptibility to infection among older patients.^34^

Due to a lack of evidence of benefit versus risk in adults aged ≥80 years, Beers criteria recommend using aspirin with caution in this age group.^9^ Therefore, 16 patients prescribed aspirin for primary prevention of cardiac events were identified as PIMs in the present study, from 112 total aspirin prescriptions.

The recommendation of Beers criteria regarding the use of NSAIDs is *‘* *to avoid chronic use unless other alternatives are not effective and the patient can take gastroprotective agent (PPI or misoprostol)*‘.^9^ This recommendation allowed the authors to identify 23 NSAIDs prescriptions as PIMs among the total of 55 taken by the study population. NSAIDs represented 10.6% of the total PIMs. A similar rate was reported by Osei *et al*.^8^

### Implications for research and practice

The present study reinforces the importance of routine medication reviews, especially in older patients. It will be useful to integrate the Beers criteria in the health informatics system as part of Clinical Decision Support to alert physicians and pharmacists to avoid any potential harm related to PIMs. The widespread dissemination of the criteria in education and training to all levels of healthcare practitioners should also take place.

The impact of this research could be significant in terms of supporting local and regional knowledge of PIMs and polypharmacy in the older patient population. Moreover, this study could pave the way for further investigations to validate the present study findings on a larger population in different settings, including those patients previously mentioned that were seen at the emergency room. Future research should evaluate interventions aimed at improving primary care follow-up and reducing the use of PIMs.

The present study showed high prevalence of PIMs, following Beers criteria, among older patients followed-up in FCM clinics in Saudi Arabia. Incremental age per 5 years, female sex, and polypharmacy were found to be associated with PIM use. The frequency of morbidities was not significantly different among patients with PIMs compared to those without PIMs, except for hypertension and osteoarthritis, which were more common in patients with PIMs than without. Long-acting sulfonylureas, PPIs, diuretics, NSAIDs, and aspirin were the most common PIMs. The present study reinforces the importance of routine medication reviews, especially in the older patient population.
